# Quantificação das Placas Coronarianas Calcificadas pela Tomografia Computadorizada do Tórax: Correlação com a Técnica do Escore de Cálcio

**DOI:** 10.36660/abc.20190235

**Published:** 2020-09-18

**Authors:** Vitor Frauches Souza, Alair Augusto Sarmet M. D. dos Santos, Claudio Tinoco Mesquita, Wolney de Andrade Martins, Gustavo Lemos Pelandre, Edson Marchiori, Marcelo Souto Nacif

**Affiliations:** 1 Complexo Hospitalar de Niterói Niterói RJ Brasil Complexo Hospitalar de Niterói – Radiologia, Niterói, RJ - Brasil; 2 Hospital Universitário Antônio Pedro Niterói RJ Brasil Hospital Universitário Antônio Pedro – Pós-Graduação em Ciências Cardiovasculares, Niterói, RJ - Brasil; 3 Universidade Federal do Rio de Janeiro Rio de Janeiro RJ Brasil Universidade Federal do Rio de Janeiro – Pós-Graduação em Radiologia, Rio de Janeiro, RJ - Brasil; 4 Universidade Federal Fluminense Niterói RJ Brasil Universidade Federal Fluminense - Pós-graduação em Ciências Cardiovasculares, Niterói, RJ - Brasil; 5 Complexo Hospitalar de Niterói Niterói RJ Brasil Complexo Hospitalar de Niterói, Niterói, RJ – Brasil

**Keywords:** Doença da Artéria Coronariana, Placa Aterosclerótica, Calcificação Vascular, Rigidez Vascular, Pontuação de Propensão, Tomografia Computadorizada por Raios-X /métodos

## Abstract

**Fundamento:**

A doença cardiovascular representa a principal causa de mortalidade no mundo. Calcificações parietais nas artérias podem ser visualizadas e quantificadas por tomografia computadorizada (TC) em estágios iniciais e subclínicos, sendo expressa em escore de cálcio (EC). Com esse número, é possível estimar o prognóstico de eventos cardiovasculares futuros.

**Objetivos:**

Correlacionar a detecção e quantificação do EC pela TC do tórax utilizando como padrão-ouro a TC cardíaca sincronizada ao eletrocardiograma.

**Métodos:**

Estudo transversal e descritivo que selecionou pacientes (n=73) consecutivos para investigação de doença arterial coronariana estável e que realizaram TC cardíaca no período de junho de 2013 a outubro de 2014. Realizado protocolo com TC do tórax e EC, em aparelho de 64 canais. Os valores de p<0,05 foram considerados estatisticamente significativos.

**Resultados:**

Na avaliação por paciente, após a transformação logarítmica a média do EC sincronizado foi de 8,7 e na TC de tórax foi de 9,4. Prevalência de doença de 49,3% (n= 36). A sensibilidade foi de 97,2% e a especificidade de 100,0%. Observou-se excelente correlação entre os métodos (r= 0,993 com p<0,001). Na avaliação por segmento, a média do EC sincronizado foi de 3,0. Já a média do EC na TC de tórax foi de 3,2. Prevalência de doença de 29,5% (n= 86), com sensibilidade de 95,3% e especificidade de 97,5%. Observou-se também excelente correlação entre os métodos (r= 0,985 com p<0,001).

**Conclusão:**

O EC sincronizado e não sincronizado têm boa correlação entre si e não mostram resultados estatisticamente diferentes. (Arq Bras Cardiol. 2020; 115(3):493-500)

## Introdução

A doença cardiovascular é a principal causa de mortalidade no mundo. Segundo a Organização Mundial da Saúde, apenas no ano de 2011, ocorreram 17 milhões de mortes decorrentes de doenças cardiovasculares, das quais 7 milhões secundárias à doença aterosclerótica coronariana (DAC) e 6,2 milhões causadas pelas doenças vasculares cerebrais.^1^ Estima-se que o número de mortes por doença cardiovascular chegará a 23,3 milhões em 2030, mantendo-se entre as principais causas de morte.^2^

O gasto anual com o tratamento da DAC é alto e envolve métodos de imagem invasivos e não-invasivos, novos medicamentos, tratamentos endovasculares e mesmo cirúrgicos, o que tem sobrecarregado cada vez mais os apertados orçamentos para a saúde. A descoberta de métodos com melhor custo-eficácia para o diagnóstico de DAC tem atraído investimentos e ajudado no rápido avanço tecnológico nesta área, contribuindo de forma exponencial para a eficácia dos tratamentos e do manejo clínico desta doença. Neste contexto, o escore de cálcio (EC) tem papel cada vez mais importante no diagnóstico da DAC.^3,4^ O EC já foi demonstrado como marcador independente de risco para eventos cardiovasculares e mortalidade cardíaca. Além disso também provém informações adicionais de prognóstico para outros marcadores de risco cardiovascular.^5^

Muitos pacientes realizam tomografia computadorizada (TC) do tórax para avaliação de diversas síndromes clínicas, como dispneia e dor torácica, assim como de possível pneumonia, massa mediastinal ou pulmonar, trauma, entre outras. Esses pacientes poderiam se beneficiariam de uma estratificação de risco para doença cardiovascular, possibilitando a ampliação de intervenções de prevenção primária e secundária. Um dado relevante é que somente nos EUA, o EC poderia potencialmente ser relatado em aproximadamente 7,1 milhões de exames de TC sem contraste realizados anualmente.^6^

Este estudo objetiva correlacionar a detecção e quantificação do EC pela TC do tórax não sincronizada ao ECG utilizando como padrão-ouro a TC cardíaca sincronizada ao ECG.

### Pacientes e métodos

Foi realizado um estudo transversal e descritivo, que selecionou pacientes em investigação de DAC e que realizaram TC cardíaca no período de junho de 2013 a outubro de 2014, de modo consecutivo. O tamanho da amostra foi por conveniência e teve relação com o número de exames realizados na unidade hospitalar no período estipulado para o trabalho. A indicação dos exames ficou a critério do médico assistente, não sendo objetivo do estudo a análise das indicações.

Os pacientes elegíveis foram submetidos a um protocolo específico para a realização da TC de tórax simples, não sincronizada, e avaliação do EC por TC cardíaca acoplada ao ECG em sessão única, sem alteração de posicionamento, com as menores doses de radiação possíveis, moduladas pelo próprio aparelho. Foram utilizados cortes de 1,0 mm de espessura na TC do tórax e para o EC cortes axiais sequenciais, sem espaçamento e com 3 mm de espessura cobrindo toda a extensão do coração, sendo este o método padrão da prática clínica.

A voltagem do tubo em todos os exames foi de 120 Kv e sua corrente variável de acordo com a modulação do aparelho, ficando entre 150 e 400 mA, seguindo os protocolos recomendados pelo fabricante e utilizados na instituição. Ambos os exames foram realizados no mesmo tomógrafo de 64 canais Sensation 64 (Siemens, Hanover, Alemanha). Todos os pacientes com frequência cardíaca acima de 70 batimentos por minuto receberam terapia betabloqueadora intravenosa com 5mg a 50mg de tartarato de metoprolol antes da aquisição das imagens.

### Cálculo do EC

A análise do cálcio coronariano foi feita por meio do EC de Agatston (aquisição sincronizada e não sincronizada ao ECG), utilizando o software *Ca Scoring* (Siemens, Hanover, Alemanha), pela análise quantitativa semiautomática. Foram considerados focos de calcificação áreas com atenuação igual ou superior a 130 unidades Hounsfield e área igual ou superior a 3 pixel e pelo menos 1mm^2^, e marcadas com sinal colorido.

A análise das artérias coronárias foi dividida em quatro ramos principais: tronco da artéria coronária esquerda (TCE), artéria descendente anterior (ADA), artéria circunflexa (ACx) e artéria coronária direita (ACD), e assim foi atribuído o escore, que é resultado do produto entre a densidade e sua área de calcificação. O EC total é resultado da soma dos escores individuais de cada região. Para cada ramo arterial foi quantificado o número de placas e o escore de Agatston.

### Análise estatística

Todas as variáveis contínuas foram expressas como média ± desvio padrão e as categóricas como número e percentual. Foram considerados os seguintes intervalos de valores de calcificação: zero (ausência de calcificação); maior do que zero e menor do que 100 (calcificação leve); maior do que 100 e menor do que 400 (calcificação moderada); maior do que 400 (calcificação grave).

Foi utilizado o teste t de *Student* para dados pareados para determinar se os resultados obtidos pelo cálculo EC eram significativamente diferentes dos obtidos pela TC do tórax na avaliação global (por paciente). Além disso, foram realizadas comparações por segmentos de cada território coronariano divididos em: TCE, ADA, ACx e ACD. As análises do EC foram tratadas com log (EC+1) para corrigir os desvios desta amostra quando necessário.

Realizou-se a análise do coeficiente de classificação de Spearman (rho) para detectar o grau de correlação entre o EC gerado pela técnica sincronizada e pela TC do tórax quanto os seguintes estratos: 0 (ausência de calcificação); 0 – 100 (calcificação leve); 100 – 400 (calcificação moderada); maior que 400 (calcificação grave).

Recorreu-se à regressão linear e ao coeficiente de correlação de Pearson (r) para avaliar a correlação entre o EC pela técnica sincronizada e não sincronizada (TC de tórax) considerando: correlação ausente se r= ZERO; fraca se r= 0,01-0,20; baixa se r= 0,21-0,40; moderada se r= 0,41-0,60; boa se r= 0,61-0,80; e excelente se r= 0,81-1,00. Também, realizamos Bland-Altman para demonstrar a variabilidade (viés) e os limites de concordância (intervalo de confiança de 95%) entre as técnicas.

Analisou-se o TCE, a ADA, a ACx e a ACD de cada paciente, totalizando 288 vasos pelo EC somado com a TC do tórax pelo observador 1. Aproximadamente 50% dos exames, ou seja, 36 pacientes e 144 segmentos, foram reavaliados pelo mesmo observador, dando maior força para os resultados encontrados. Também foram realizadas análises por um segundo observador independente, de maneira totalmente cega ao primeiro observador. Além disso, o observador 2 repetiu esta análise após o período de 3 meses de forma totalmente cega à primeira análise. A concordância intraobservador e interobservador foi obtida usando-se a análise da confiabilidade das médias do coeficiente de correlação intraclasse (CCI <0,40 fraca concordância; CCI = 0,40-0,59 moderada concordância; CCI = 0,60-0,74 boa concordância; CCI = 0,75-1,00 excelente concordância). Reiteramos que as análises foram totalmente cegas e baseadas na experiência de ambos os observadores. Vale frisar que o observador 1 possui 2 anos de experiência e o observador 2 acima de 12 anos.

Análise de curva característica de operação do receptor (receiver operating characteristic, ROC) foi utilizada para identificar o desempenho diagnóstico da TC de tórax (não sincronizada) para predizer o EC (sincronizado) por paciente e por segmento. Isto foi realizado com uso dos grupos 0 – 100 (calcificação leve); 101 – 400 (calcificação moderada); maior que 400 (calcificação grave) como marcadores substitutos de “verdadeiro positivo” nesta população, em comparação com o grupo com 0 (ausência de calcificação) como o “verdadeiro negativo” (AUC ≥0,5 e <0,7= ajuste pobre, AUC ≥0,7 e <0,9= bom ajuste e AUC ≥0,9 e 1,0= excelente ajuste).

Para a análise estatística foi utilizado o MedCalc®, versão 17.8 (MedCalc Software bvba, Ostend, Bélgica). Os valores de p<0,05, bicaudais, foram considerados estatisticamente significativos.

O estudo foi aprovado pela comissão de ética da instituição sede do estudo, sob o número 771.854. O estudo seguiu os preceitos éticos determinados pela Declaração de Helsinque. Todos os pacientes participantes assinaram o termo de consentimento livre e esclarecido, tendo sido garantidas a retirada do consentimento e a exclusão do estudo a qualquer momento, a pedido do indivíduo ou de seu responsável, sem prejuízo à assistência, confidencialidade, sigilo ou privacidade.

## Resultados

De um total de 82 pacientes, foram incluídos no estudo 73 pacientes, 37 (51,4%) do sexo masculino, com a média de idade de 58,9±13,1 anos, entre junho de 2013 e outubro de 2014. Durante a amostragem foram excluídos do estudo nove pacientes, sendo quatro pela presença de *stents* coronarianos, um por enxerto de mamária, e outros quatro por dificuldades técnicas tais como artefatos de movimento que impediram a análise das imagens e problemas de envio das sequências adquiridas para o servidor de imagens.

### Análise por paciente

Após a transformação logarítmica, observou-se que o método não sincronizado (TC de tórax) superestima discretamente os valores obtidos pelo sincronizado, com médias de 9,4 e 8,7, respectivamente.

**Figura 1:**
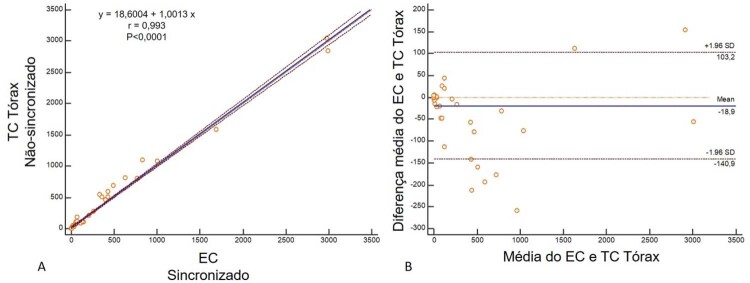
Análise por paciente. (A) Regressão linear demonstrando a correlação entre o método de tomografia computadorizada de tórax não sincronizada com ecocardiografia (ECG) e o método de tomografia computadorizada cardíaca sincronizada com ECG. (B) Análise de Bland-Altman com os dados obtidos por ambos os métodos. A diferença média é representada pela linha azul e o limite de concordância pelo traçado vermelho; TC: tomografia computadorizada do tórax; EC: escore de cálcio.

Foi averiguada excelente correlação entre os métodos. Achados estes que podem ser observados na Figura 1.

Dos 73 pacientes estudados, 37 apresentaram EC (sincronizado) igual a zero; 18 com EC entre 0 – 100; sete pacientes com EC entre 100 – 400; e 11 pacientes com EC > 400. Pela TC de tórax (não sincronizada) o EC igual a zero (ausência de calcificação) foi encontrado em 35 indivíduos; 19 apresentaram EC entre 0 – 100 (calcificação leve); 6 tiveram o EC entre 100 – 400 (calcificação moderada); e 13 com EC > 400 (calcificação grave). A correlação do coeficiente de classificação de Spearman (rho) para classificação dos grupos acima foi de 0,96. A Tabela 1 sintetiza os achados da análise por paciente.

**Tabela 1:** – Distribuição dos pacientes pela classificação de escore de cálcio pelas técnicas de tomografia computadorizada sincronizada e não sincronizada com ecocardiografia, na análise por paciente (n=73)

	Técnica sincronizada	Escore de Cálcio	Técnica não sincronizada	Escore de cálcio
Grupos	N (%)	Média e DP	N (%)	Média e DP
0	37 (50,7)	0	35 (47,9)	0
0 a 100	18 (24,7)	24,3 ± 24,7	19 (26,0)	26,0 ± 34,0
100 a 400	7 (9,6)	222,4 ± 99,8	6 (8,2)	166,3 ± 71,5
>400	11 (15,1)	1150,7 ± 980,2	13 (17,8)	1118,2 ± 867,7

DP: desvio padrão.

### Análise segmentar

A média do EC sincronizado (método padrão) na análise segmentar foi de 50,1±179,7 Agatston e sua média após transformação logarítmica foi de 3,0 Agatston com intervalo de confiança de 95% de 2,4 a 3,8. Já a média do EC na TC de tórax foi de 54,9±184,7 Agatston e sua média após transformação logarítmica foi de 3,2 Agatston com intervalo de confiança de 95% de 2,5 a 4,1.

**Figura 2:**
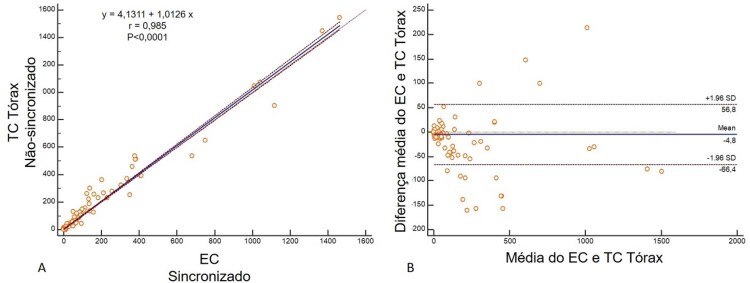
– Análise por segmento. (A) Regressão linear demonstrando a correlação entre o método de tomografia computadorizada de tórax não sincronizada com ecocardiografia (ECG) e o método de tomografia computadorizada cardíaca sincronizada com ECG. (B) Análise de Bland-Altman com os dados obtidos por ambos os métodos. A diferença média é representada pela linha azul e o limite de concordância pelo traçado vermelho.

Na Figura 2, pode-se observar que foi encontrada excelente correlação entre os métodos.

Na análise segmentar, 292 segmentos foram incluídos, sendo que 206 apresentaram EC igual a zero; 56 com EC entre 0 – 100; 21 segmentos com EC entre 100 – 400; nove segmentos com EC > 400. Pela TC de tórax não sincronizada, o EC igual a zero foi encontrado em 197; em 59 segmentos foi encontrado EC entre 0 – 100; EC entre 100 – 400 em 25 segmentos; e 11 com EC > 400. A correlação do coeficiente de classificação de Spearman (rho) para classificação segmentar dos grupos anteriormente descritos foi de 0,92. Os achados da análise segmentar estão apresentados na Tabela 2.

**Tabela 2:** – Distribuição dos pacientes pela classificação de escore de cálcio pelas técnicas de tomografia computadorizada sincronizada e não sincronizada com ecocardiografia, na análise por segmento (n=292)

	Técnica sincronizada	Escore de Cálcio	Técnica não sincronizada	Escore de cálcio
Grupos	N (%)	Média e DP	N (%)	Média e DP
0	206 (70,5)	0	197 (67,5)	0
0 a 100	56 (19,2)	28,6 ± 29,9	59 (20,2)	20,1 ± 22,6
100 a 400	21 (7,2)	228,4 ± 101,1	25 (8,6)	225,5 ± 93,0
>400	9 (3,1)	917,5 ± 381,7	11 (3,8)	838,1 ± 393,5


*DP: desvio padrão.*

### Correlação segmentar entre os métodos

A média do EC pelo método sincronizado com ECG foi 200,7 Agatston, e sua distribuição nas coronárias da seguinte forma: TCE 6,9; ADA 88,7; ACx 26,4 e ACD 88,6. Já a média do EC na TC de tórax foi 219,5 e sua distribuição nas coronárias: TCE 8,4; ADA 85,4; ACx 29,1 e ACD 96,6. Tais dados podem ser mais bem visualizados na Tabela 3.

**Tabela 3:** – Correlação por segmentos coronarianos entre o escore de cálcio obtido pelas técnicas de tomografia computadorizada (TC) sincronizada (TC cardíaca) e não sincronizada (TC de tórax) com ecocardiografia

	Escore de cálcio	TC de tórax	Pearson	Teste t	
	Média e DP	Média e DP	r (95%IC)	Teste t (p)	N
TCE	6,9 ± 23,4	8,4 ± 27,3	0,90 (0,85 a 0,93)	0,25	73
ADA	88,7 ± 278,5	85,4 ± 198,5	0,97 (0,96 a 0,95)	0,85	73
ACx	26,4 ± 75,5	29,1 ± 78,7	0,98 (0,96 a 0,98)	0,14	73
ACD	88,6 ± 278,5	96,6 ± 293,1	0,99 (0,98 a 0,99)	0,08	73

*TCE: tronco da coronária esquerda; ADA: artéria descendente anterior; ACx: artéria circunflexa; ACD: artéria coronária direita.*

### Prevalência, especificidade e sensibilidade

**Figura 3:**
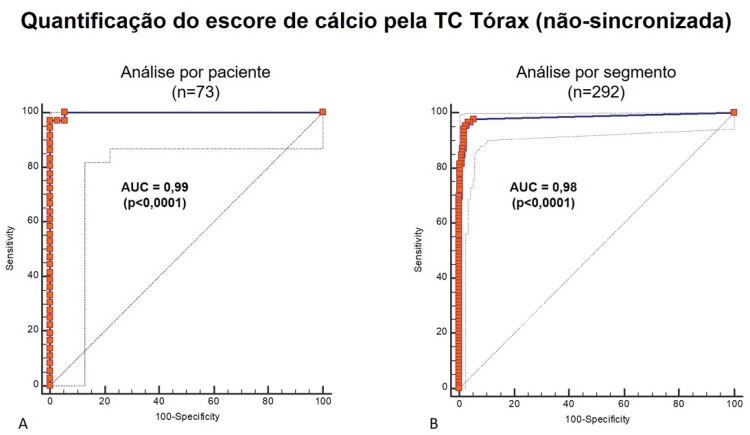
– Análise da curva ROC (receiver operating characteristic) com quantificação da área sob a curva utilizando a tomografia computadorizada do tórax para predizer placa calcificada detectada pelo escore de cálcio; (A) análise por paciente; B) análise por segmento.

Os dados encontrados de especificidade e sensibilidade tanto para a análise por paciente quanto por segmento estão dispostos na Figura 3.

Na análise por paciente (n= 73), com uma prevalência de doença de 49,3% (n= 36), a TC de tórax foi capaz de identificar os pacientes com placas calcificadas com uma área sob a curva (ROC_AUC) de 0,99 com intervalo de confiança de 95% de 0,99 a 1,00 (p<0,0001). A sensibilidade foi de 97,2% e a especificidade de 100,0%.

Já na avaliação segmentar (n= 292), com uma prevalência de doença de 29,5% (n= 86), a TC de tórax foi capaz de identificar os segmentos com placas calcificadas com uma área sob a curva (ROC_AUC) de 0,98 com intervalo de confiança de 95% de 0,96 a 1,00 (p<0,0001). A sensibilidade foi de 95,3% e a especificidade de 97,5%.

**Figura 4:**
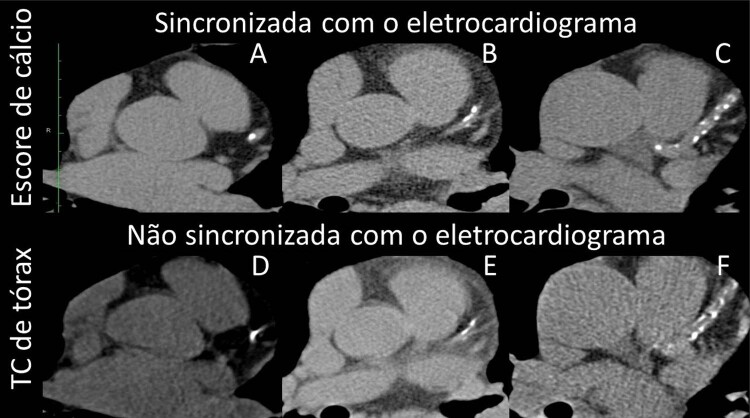
– Exemplos de quantificação pela técnica do escore de cálcio (A, B e C), sem movimento da coronária. Mesmos pacientes com a quantificação pela tomografia computadorizada de tórax (D, E e F) com um certo grau de movimento da coronária. Observar as placas na descendente anterior: o primeiro caso com pequena calcificação (A e D); um segundo caso com duas calcificações (B e E) e um terceiro caso com múltiplas placas calcificadas (C e F).

A Figura 4 demonstra três exemplos diferentes de calcificação na artéria descendente anterior, caracterizados pelo EC e pela TC do tórax.

### Concordância intraobservador e interobservador

Excelente concordância intraobservador e interobservador foi demonstrada na quantificação das placas calcificadas pela técnica do EC e pela técnica da TC de tórax, com CCI > 0,99 para todos.

## Discussão

Nos últimos anos o rastreio de pacientes assintomáticos para a descoberta de doença cardiovascular em estágios iniciais tem ganhado grande relevância socioepidemiológica e causado controvérsia no âmbito dos estudos científicos.^3,7-9^

Indubitavelmente a grande quantidade de exames de TC de tórax realizadas por outros propósitos pode auxiliar no acompanhamento cardiovascular de pacientes assintomáticos com o fornecimento de informações clínicas relevantes, evitando-se a repetição de exames para esse fim ou até mesmo selecionando os pacientes que merecem o tratamento adequado.

Desde seu advento, a TC demonstrou ser um ótimo método para detecção de câncer de pulmão e de outras doenças pulmonares. Com os últimos avanços, foi possível a realização de exames de coração sincronizados ao ECG, possibilitando a quantificação de cálcio coronariano e, por conseguinte, estratificação do risco cardiovascular. Nesse contexto, a realização de um único exame de TC em que fosse possível avaliar a presença de tumores pulmonares e o risco cardiovascular teria uma significativa relevância clínica.^10-15^

A TC sincronizada ao ECG ainda é o método referência para identificação e quantificação do cálcio coronariano, com validação estabelecida. No entanto, estudos clássicos com TC do tórax não sincronizada ao ECG também mostraram que a identificação visual (não quantitativa) do cálcio coronariano fornece informações clínicas relevantes.^12,15-17^ Desta forma, é evidente que a avaliação quantitativa do EC na aquisição da TC de tórax pode revelar dados ainda mais importantes e fidedignos.

O grande avanço tecnológico dos tomógrafos nos últimos 20 anos permitiu uma notória melhora na avaliação das calcificações coronarianas nos exames não sincronizados ao ECG, pois os cortes mais finos e a rapidez na aquisição dos dados reduziram significativamente os artefatos produzidos por movimentos cardíacos e por efeito de volume parcial.^18^

Nesse espectro, este trabalho objetivou demostrar a relação entre os valores de EC encontrados na tomografia cardíaca sincronizada ao ECG em aparelho de 64 canais de detectores com aqueles vistos em exames do tórax com baixa dose de radiação, realizados no mesmo aparelho, a fim de possibilitar um aumento de informações radiológicas e clínicas nas TCs de tórax realizadas rotineiramente nos hospitais e clínicas.

Foi possível detectar e quantificar o EC pela TC de tórax não sincronizada e, quando se comparou com a técnica padrão-ouro, o EC sincronizado, os resultados foram excelentes. Porém, com a transformação logarítmica dos valores encontrados, observou-se que a TC de tórax superestima discretamente os valores obtidos pelo método de referência (p= 0,0012), uma vez que está mais suscetível às alterações causadas por artefatos de movimento respiratório e pelos batimentos cardíacos.

Todas as correlações de Pearson foram superiores a 0,90 (p<0,0001) na análise por paciente ou segmentar. Há variabilidades entre os métodos, com viés muito pequeno (4,1 Agatston) e achados inferiores a 3,2% da diferença média entre as técnicas, e esses resultados não são de importância clínica. Esses achados vão ao encontro do estudo de Budoff et al.,^19^ demonstrando excelente correlação entre os dois métodos.

A TC de tórax foi capaz de classificar a população conforme as diretrizes atuais, com excelente correlação e sem diferença estatística significativa quando comparada ao método ouro por territórios coronarianos. Os valores encontrados para a correlação de Pearson nesta avaliação variaram entre 0,90 e 0,99 e o p do teste T de *student*, entre 0,08 e 0,85, conforme os dados disponíveis na Tabela 3.

Em apenas dois casos foram observados falsos positivos, no qual o EC total foi zero na técnica padrão ouro e 0,3 e 0,6 Agatston na TC de tórax. Tal achado pode ter duas explicações plausíveis, a primeira devido à presença de artefatos inerentes ao método e que foram considerados como calcificação pelo *software*, visto a diminuta carga cálcica nesses dois achados, e a segunda relacionada aos cortes mais finos da TC de tórax (1 mm) comparados aos do EC (3 mm); desta forma, pequeninas placas antes não identificadas pelo método ouro, poderiam ser identificadas na TC de tórax. Isso abre um questionamento sobre a espessura de corte da técnica padrão do EC ser a mesma desde sua criação por Agatston no início dos anos 90, quando ainda não eram disponíveis aparelhos com tecnologia para cortes mais finos.

Desta forma, incentiva-se o uso, como forma alternativa, da TC de tórax não sincronizada para a detecção do EC. Esse método possui capacidade diagnóstica semelhante ao padrão-ouro, atingindo excelente sensibilidade e especificidade, com área sob a curva superior a 0,98 (p<0,0001), demonstrando assim, sensibilidade de 97,2% e especificidade de 100,0% para a análise por paciente e sensibilidade de 95,3% e especificidade de 97,5% quanto feito por segmento coronariano. Pelandré et al.^20^ demonstraram a capacidade de utilização do método em um estudo semelhante, com excelente acurácia utilizando software dedicado ou análise visual.

Neste contexto, a sociedade de TC cardiovascular (SCCT – *Society for Cardiovascular Computed Tomography*) desenvolveu uma diretriz para utilização da TC de tórax na avaliação de placas calcificadas coronarianas, demonstrando a importância da descrição desses achados de forma quantitativa e qualitativa, além da topografia destas placas.^21^

Pelo exposto, demonstra-se o quão importante é para o radiologista a descrição por completo da presença ou não de cálcio nos estudos tomográficos do tórax, e quando possível sua quantificação, visto que o estudo comprovou que a capacidade diagnóstica é excelente mesmo frente a diferença entre os métodos.

### Limitação do estudo

A limitação deste estudo foi em virtude da necessidade de administração de betabloqueador adrenérgico, prática que não é rotineira nas TC de tórax. Acreditamos que pacientes com frequência cardíaca elevada terão maiores artefatos nas imagens não trigadas. Também consideramos de extrema importância a aplicabilidade destes resultados em diferentes centros com diferentes tomógrafos, porém os nossos resultados não devem ser extrapolados sem uma testagem inicial para as outras tecnologias.

## Conclusão

O presente estudo demonstrou excelente correlação entre a quantificação das placas calcificadas pela TC de tórax não sincronizada ao ECG quando comparada ao EC em estudos com a utilização de betabloqueadores.

De posse dos resultados deste estudo, incentiva-se o uso, como forma alternativa, da TC de tórax não sincronizada para a detecção do EC, a fim de auxiliar no acompanhamento e estratificação do risco de pacientes que muitas vezes não tem acesso a uma tomografia cardíaca.
