# Supplementary material for: Quantificação das Placas Coronarianas Calcificadas pela Tomografia Computadorizada do Tórax: Correlação com a Técnica do Escore de Cálcio
Source: Arq Bras Cardiol. 2020 Sep 18;115(3):493–500. [Article in Portuguese] doi: 10.36660/abc.20190235 (PMC9363106; doi:10.36660/abc.20190235)
Supplement: Translation

# Translation: Quantification of Calcified Coronary Plaques by Chest Computed Tomography: Correlation with the Calcium Score Technique

**Authors:** Vitor Frauches Souza, Alair Augusto Sarmet M. D. dos Santos, Claudio Tinoco Mesquita, Wolney de Andrade Martins, Gustavo Lemos Pelandre, Edson Marchiori, Marcelo Souto Nacif

**Affiliations:** 1 Brasil Complexo Hospitalar de Niterói – Radiologia, Niterói, RJ - Brasil; 2 Brazil Hospital Universitário Antônio Pedro – Pós-Graduação em Ciências Cardiovasculares, Niterói, RJ - Brazil; 3 Brazil Universidade Federal do Rio de Janeiro – Pós-Graduação em Radiologia, Rio de Janeiro, RJ - Brazil; 4 Brazil Universidade Federal Fluminense - Pós-graduação em Ciências Cardiovasculares, Niterói, RJ - Brazil; 5 Brazil Complexo Hospitalar de Niterói, Niterói, RJ – Brazil

**Keywords:** Coronary Artery Disease, Plaque,Atherosclerotic, Vascular Calcification, Vascular Stiffness, Tomography, X-Ray/ methods

## Abstract

**Background:**

Cardiovascular disease is the leading cause of mortality in the world. Parietal calcifications of the arteries may be visualized and quantified at initial and subclinical states by computed tomography (CT), and expressed as calcium score (CS). It is possible to estimate the prognosis of future cardiovascular events using this score.

**Objectives:**

To correlate the detection and quantification of the CS obtained by chest CT with that obtained by electrocardiography (ECG)-synchronized cardiac computed tomography (the gold-standard).

**Method:**

Cross-sectional, descriptive study of 73 consecutive patients in investigation for coronary artery disease who underwent cardiac CT between June 2013 and October 2014. Chest computed tomography and CS protocols were performed in a 64-channel TC scanner. P-values <0.05 were considered statistically significant.

**Results:**

In the per-patient analysis, after logarithmic transformation, mean CS was 8.7 and 9.4 by the ECG-synchronized method and chest CT, respectively. The prevalence of disease was 49.3% (n=36), with a sensitivity of 97.2% and specificity of 100.0%. There was an excellent correlation between the methods (r= 0.993, p<0.001). In the per-segment analysis, after logarithmic transformation, mean CS was 3.0 and 3.2 by the ECG-synchronized method and chest CT, respectively. The prevalence of disease was 29.5% (n=86), with a sensitivity of 95.3% and specificity of 97.5%. There was an excellent correlation between the methods (r= 0.985, p<0.001).

**Conclusion:**

ECG-synchronized CT is well correlated with the non-ECG-synchronized CT for CS determination, without statistical difference between the methods. (Arq Bras Cardiol. 2020; 115(3):493-500)

## Introduction

Cardiovascular disease is the leading cause of death in the world. According to the World Health Organization, 17 million people died from cardiovascular diseases only in the year 2011; 7 million of them from coronary artery disease (CAD) and 6.2 million from cerebrovascular diseases.^1^ It is estimated that the number of deaths from cardiovascular disease will achieve 23.3 million by 2030, and continue among the main causes of death.^2^

The annual cost of treatment of CAD is high and involves invasive and non-invasive imaging tests, new medications, endovascular and even surgical treatment, which has increasingly affected the limited health budget. The development of new methods with high cost-effectiveness for CAD diagnosis has attracted investments and contributed to the rapid technological advances in this field and to the improvement of treatment efficacy and clinical management of the disease. In this context, calcium score (CS) has played an important role in the diagnosis of CAD.^3,4^ It has already been shown as an independent risk marker for cardiovascular events and cardiac mortality. Besides, the score provides additional prognostic information to other markers of cardiovascular risk.^5^

Many patients undergo chest computed tomography (CT) for assessment of many clinical syndromes, such as dyspnea and chest pain, and of possible pneumonia, mediastinal or pulmonary mass, trauma, among others. These patients could benefit from a risk stratification for cardiovascular disease, allowing the performance of a wider variety of primary and secondary preventive interventions. Only in the USA, CS may potentially be reported in approximately 7.1 million CT tests without contrast performed annually.^6^

The present study aimed to correlate the detection and quantification of CS by non-ECG-synchronized chest CT, using the ECG-synchronized cardiac CT as the gold standard.

### Patients and methods

This was a descriptive, cross-sectional study of consecutive patients in investigation for CAD, who underwent cardiac CT between June 2013 and October 2014. A convenience sample size was used, which was related to the number of tests conducted in the hospital during the period of study. Indications of the CT test was at the discretion of the assistant physician and was not analyzed in the study.

Eligible patients underwent a protocol of simple, non-synchronized chest CT, and evaluation of CS by ECG-synchronized CT, in the same position and in only one session. Radiation doses were as low as possible, modulated by the CT device. In chest CT, 1.0 mm slice thickness was used, and quantification of CS was performed according to the standard method used in clinical practice, using contiguous axial slices with a thickness of 3 mm, covering the entire heart.

The voltage of the CT x-ray tube was of 120 Kv, and the tube current depended the scanner modulation and varied from 150 to 400 mA, according to protocols recommended by the manufacturer, used in the institution. Both tests were carried out using a 64-channel scanner (Siemens, Hanover, Germany). All patients with a heart rate over 70 beats per minute received intravenous beta-blocker therapy (5-50mg metoprolol tartrate) before image acquisition.

### Calculation of CS

Analysis of coronary artery calcium was made by the Agatston score (ECG-synchronized and non-ECG-synchronized), using the Ca Scoring software (Siemens, Hanover, Germany), by semiautomated quantitative analysis. Areas of calcification were defined as areas with attenuation above 130 Hounsfield units, area equal to or greater than 3 pixels and 1mm^2^, and colored overlay.

Analysis of coronary arteries was divided into four main branches: the left coronary artery (LCA), the left anterior descending artery (LAD), the circumflex artery (Cx), the right coronary artery (RCA); CS, calculated by multiplying the density by the area of calcification, was attributed accordingly. The total score resulted from the sum of the score in each of these branches. For each arterial branch, the number of plaques was quantified and the Agatston score calculated.

### Statistical analysis

All continuous variables were expressed as mean and standard deviation and categorical variables as number and percentages. The following calcification intervals were considered for analyses: zero (no calcification); greater than zero and lower than 100 (mild calcification); greater than 100 and lower than 400 (moderate calcification); greater than 400 (severe calcification). The paired t-test was used for paired data to determine whether results obtained from CS calculation were significantly different from those obtained by chest CT and global assessment (per patient). In addition, comparisons were made between the segments of each coronary territory, divided into LCA, LAD, Cx and RCA. CS analyses were treated with log (CS + 1) to correct for deviation of this sample, as appropriate.

The Spearman’s rank correlation coefficient (rho) was used to determine the degree of correlation of coronary artery calcification between the ECG-synchronized cardiac CT and the chest CT for the following strata: 0 (no calcification); 0-100 (mild calcification); 100 – 400 (moderate calcification); > 400 (severe calcification).

The liner regression and the Pearson correlation coefficient (r) were used to assess the correlation of CS between the ECG-synchronized cardiac CT and the chest CT. A r=ZERO indicated no correlation; r=0.01-0.20 slight correlation; r=0.21-0.40 poor correlation; r=0.41-0.60 moderate correlation; r=0.61-0.80 good correlation, and r=0.81-1.00 indicated excellent correlation. Also, the Bland-Altman plot was used to show the variability (bias) and the agreement limits (95% confidence interval) between the methods.

Of each patient, the LCA, LAD, Cx, and the RCA were examined, giving a total of 288 vessels by the CS and the chest CT analyzed by the observer 1. Approximately 50% of the tests, *i.e.*, 36 patients and 144 segments, were reviewed by the same observer, thereby increasing the strength of the results. Analyses were also made by a second independent observer, blinded for the first observer, to evaluate variability between observers. In addition, observer 2 repeated this analysis after three months, blinded for the first analysis. Intra- and interobserver agreement was obtained by assessing reliability of mean intraclass correlation coefficient (ICC) (ICC<0.40 = poor agreement; ICC 0.40-0.59 moderate agreement; ICC: 0.60-0.74 good agreement; ICC: 0.75-1.00 excellent agreement). Again, all analyses were blinded and based on the experience of the observers. Observer 1 has two years of experience, the other has more than 12 years.

Receiver operating characteristic (ROC) curve analysis was made to determine the diagnostic performance of chest CT (non-ECG-synchronized) in predicting the (synchronized) CS per patient and per segment. In this analysis, the groups 0-100 (mild calcification); 101 – 400 (moderate calcification); > 400 (severe calcification) were used as surrogates for “true-positive” in this population, in comparison with “zero” (no calcification) as “false-negative” (AUC ≥0.5 and <0.7= poor adjustment; AUC ≥0.7 and <0.9= good adjustment and AUC ≥0.9 and 1.0= excellent adjustment).

The MedCalc®, version 17.8 (MedCalc Software bvba, Ostend, Belgium) was used for the statistical analysis. Two-tail p-values <0.05 were considered statistically significant.

The study was approved by the ethics committee of the institution (approval number 771.854). The study was conducted following the Declaration of Helsinki. All participants signed the informed consent form, and the patient or guardian had the right to withdraw consent or participation at any time, without jeopardizing their right of access to other health services, to confidentiality and privacy.

## Results

Between June 2013 and October 2014, 73 out of 82 patients were included in the study, 37 (51.4%) men, mean age of 58.9 ± 13.1 years. Nine patients were excluded, four due to the presence of coronary stents, one for the presence of mammary graft, and four for technical difficulties, such as motion artifacts that made the analysis of the images impossible and problems in sending the sequences to the image server.

### Per-patient analysis

After logarithmic transformation, we found that the non-synchronized chest CT slightly overestimate the ECG-synchronized CT method, with means of 9.4 and 8.7, respectively.

An excellent correlation was found between the methods. These findings can be seen in Figure 1.

**Figure 1:** Per-patient analysis. (A) Linear regression showing the correlation between the non-echocardiography (ECG)-synchronized chest computed tomography and the ECG-synchronized cardiac computed tomography. (B) Bland-Altman analysis of data obtained from both methods; mean difference (blue line) and agreement limits (red tracing); CT: computed tomography; CS: calcium score.

Of 73 patients, a CS (by the ECG-synchronized method) of zero was found in 37 patients, 0-100 in 18 patients, 100-400 in seven, and >400 in 11 patients. Using chest CT (non-ECG-synchronized) CS was zero in 35 patients, 0-100 (mild calcification) in 19 patients, 100-400 (moderate calcification) in six, and >400 (severe calcification) in 13 patients. Spearman coefficient correlation between the methods for the CS classification was greater than 0.96. Results of the per-patient analysis are summarized in Table 1.

**Table 1:** – Distribution of patients by calcium score classification by the echocardiography (ECG)-synchronized and the non-ECG synchronized methods in per-patient analysis (n=73)

	ECG-synchronized	Calcium score	Non-ECG-synchronized	Calcium score
Groups	N (%)	Mean and SD	N (%)	Mean and SD
0	37 (50.7)	0	35 (47.9)	0
0 - 100	18 (24.7)	24.3 ± 24.7	19 (26.0)	26.0 ± 34.0
100 - 400	7 (9.6)	222.4 ± 99.8	6 (8.2)	166.3 ± 71.5
>400	11 (15.1)	1150.7 ± 980.2	13 (17.8)	1118.2 ± 867.7

*SD: standard deviation*

### Per-segment analysis

Mean CS by the gold standard ECG-synchronized method in the per-segment analysis was 50.1 ± 179.7 Agatston, with a mean of 3.0 Agatston after logarithmic transformation and 95% confidence interval of 2.4-3.8. By chest CT, mean CS was 54.9 ± 184.7 Agatston, with a mean of 3.2 after logarithmic transformation and 95% confidence interval of 2.4-3.8. There was an excellent correlation between the methods, as shown in Figure 2.

**Figure 2:** – Per-segment analysis. (A) Linear regression showing the correlation between the non-echocardiography (ECG)-synchronized chest computed tomography and the ECG-synchronized cardiac computed tomography. (B) Bland-Altman analysis of data obtained from both methods; mean difference (blue line) and agreement limits (red tracing); CT: computed tomography; CS: calcium score.

A total of 292 segments was included in the per-segment analysis; 206 had a CS equal to 0; 56 had a CS of 0-100; 21 segments had a CS of 100-400; nine segments a CS >400. By non-ECG-synchronized chest TC, 197 segments had a CS equal to 0; 59 segments had a CS of 0-100; 25 segments had a CS of 100-400; and 11 segments a CS >400. Spearman coefficient correlation between the methods for the CS classification was 0.92. Results of the per-segment analysis are summarized in Table 2.

**Table 2:** – Distribution of patients by calcium score classification by the echocardiography (ECG)-synchronized and the non-ECG synchronized methods in per-segment analysis (n=292)

	ECG-synchronized	Calcium score	Non-ECG-synchronized	Calcium score
Groups	N (%)	Mean and SD	N (%)	Mean and SD
0	206 (70.5)	0	197 (67.5)	0
0 - 100	56 (19.2)	28.6 ± 29.9	59 (20.2)	20.1 ± 22.6
100 - 400	21 (7.2)	228.4 ± 101.1	25 (8.6)	225.5 ± 93.0
>400	9 (3.1)	917.5 ± 381.7	11 (3.8)	838.1 ± 393.5

*SD: standard deviation*

### Segment correlation between the methods

Mean CS by the ECG-synchronized method was 200.7 Agatston, distributed among the coronary arteries as follows: 6.9 in the LCA, 88.7 in the LAD, 26.4 in the Cx, and 88.6 in the RCA. Mean CS by chest CT was 219.5 Agatston, distributed in the coronary arteries as follows: 8.4 in the LCA, 85.4 in the LAD, 29.1 in the Cx, and 96.6 in the RCA (Table 3).

**Table 3:** – Correlation between calcium score determined by the echocardiography (ECG)-synchronized and calcium score determined by the non-ECG synchronized method

	Calcium score (Cardiac computed tomography)	Chest computed tomography	Pearson	T Test	
	Mean and SD	Mean and SD	r (95%IC)	T test (p)	N
LCA	6.9 ± 23.4	8.4 ± 27.3	0.90 (0.85 a 0.93)	0.25	73
LAD	88.7 ± 278.5	85.4 ± 198.5	0.97 (0.96 a 0.95)	0.85	73
Cx	26.4 ± 75.5	29.1 ± 78.7	0.98 (0.96 a 0.98)	0.14	73
RCA	88.6 ± 278.5	96.6 ± 293.1	0.99 (0.98 a 0.99)	0.08	73

*LCA: left coronary artery, LAD: left anterior descending artery, Cx: circumflex artery (Cx); RCA: right coronary artery.*

### Prevalence, specificity and sensitivity

Per-patient and per-segment specificity and sensitivity are described in Figure 3. In the per-patient analysis (n=73), the prevalence of disease was 49.3% (n=36), and chest CT detected patients with calcified areas, with area under the ROC curve of 0.99 and 95% confidence o=interval of 0.99-1.00 (p<0.0001). A sensitivity of 97.2% and a specificity of 100.0% were found.

**Figure 3:** – ROC (receiver operating characteristic) curve analysis and area under the ROC curve by chest computed tomography to predict calcified plaque detected by the calcium score; (A) per-patient analysis, B) per-segment analysis.

In the per-segment analysis (n=292), the prevalence of disease was 29.5% (n=86), and chest CT detected patients with calcified areas, with area under the ROC curve of 0.98 and 95% confidence o=interval of 0.96-1.00 (p<0.0001). A sensitivity of 95.3% and a specificity of 97,5% were found.

Figure 4 illustrates three different examples of calcification in the anterior descending artery, characterized by the CS and chest CT.

**Figure 4:** – Examples of quantification by the calcium score technique (A, B, and C), without cardiac motion. Same patients using chest computed tomography (D, E and F), with some degree of cardiac motion. Small calcification (A and D), two calcifications (B and E) and multiple calcified plaques (C and F) in the anterior descending artery

### Intraobserver and interobserver agreement

An excellent intraobserver and interobserver agreement was observed in the quantification of calcified plaques by the CS technique and by chest CT, with an ICC >0.99.

## Discussion

In the last years, the screening for asymptomatic patients in the investigation of cardiovascular disease at initial stages has gained importance in social and epidemiological context and been a subject of controversy.^3,7-9^

Certainly, the large amount of chest CT tests performed for other reasons can help in the follow-up of asymptomatic cardiovascular patients, providing relevant clinical information, preventing the repetition of tests for this purpose and even selecting the patients to receive the most appropriate treatment.

Since its advent, CT has been shown to be an excellent method for detection of lung cancer and other pulmonary diseases. With recent advances, cardiac tests have been carried out synchronized with ECG, allowing the quantification of coronary artery calcium and cardiovascular risk stratification. The possibility of detecting lung tumors and assessing cardiovascular risk by one single CT test would be of high clinical relevance.^10-15^

ECG-synchronized CT is still the reference method for detection and quantification of coronary calcium, with well-established validation. However, classic studies with non-ECG-synchronized chest CT have also shown that visual identification (*i.e*., not quantitative) of coronary calcium can provide important information.^12,15-17^ Thus, it is evident that the quantitative evaluation of CS by chest CT could reveal even more significant and reliable data.

The great technological advances in CT scanners in the last 20 years have enabled considerable improvement in the assessment of coronary artery calcium by non-ECG-synchronized tests, since thinner slices and faster acquisition of data significantly reduced the cardiac motion and partial volume artifacts.^18^

In this scenario, the present study aimed to show the relationship between CS values obtained by ECG-synchronized cardiac CT using a 64-slice CT scanner and those obtained by low-dose chest CT, performed by the same device, so as to the chest CT tests, routinely performed in clinics and hospitals, would provide a greater amount of radiological and clinical information.

CS could be successfully detected by the non-ECG-synchronized chest CT and, with excellent performance as compared with the gold-standard technique. However, after logarithmic transformation, we found that chest CT slightly overestimates the values obtained by the reference method (p=0.0012), since it is more susceptible to changes caused by respiratory motion artifacts and heart beats.

All Pearson correlations in per-patient and per-segment analyses were higher than 0.90 (p<0.0001). There is variability between the methods, but with minimum bias (4.1 Agatston) and mean difference between the techniques lower than 3.2%, without clinical significance. These findings are in accordance with the study by Budoff et al.,^19^ which showed an excellent correlation between the methods.

Chest CT was able to classify the population by CS according to current guidelines, with excellent correlation and no statistically significant difference as compared with the gold-standard method by coronary territory. Pearson correlation values varied from 0.90 to 0.99 and p-values of the Student’s t-test varied from 0.08 to 0.85, as can be seen in Table 3.

False positive results were found in only two patients, who showed a CS of zero by the gold-standard method and of 0.3 and 0.6 Agatston by chest CT. There are two plausible explanations for this finding: the first is the presence of artifacts, inherent to the method, that may have been considered as “calcification” by the software program due to the very low calcium load in these two cases; the second is related to the thinner slices (1 mm) of chest CT compared with CS (3 mm), so as tinny plaques not detected by the gold-standard method may have been detected by the chest CT. This raises a question about the slice thickness adopted for CS determination using the gold-standard method, developed by Agatston in the beginning of the 1990’s, when CT scanners with technology for thinner slices were not available.

Therefore, the use of non-ECG-synchronized CT, as an alternative technique for CS determination should be encouraged. This method has a diagnostic performance similar to the gold-standard method, with excellent sensitivity and specificity, and area under the curve greater than 0.98 (p<0.0001) – sensitivity of 97.2% and specificity of 100.0% for per-patient analysis and sensitivity of 95.3% and specificity of 97.5% for per-segment analysis. In a similar study, Pelandré et al.^20^ showed the performance of the method, with excellent accuracy using a dedicated software or by visual analysis.

In this context, the Society for Cardiovascular Computed Tomography developed a guideline on the use of chest CT in the assessment of coronary calcified plaques, indicating the importance of quantitative and qualitative analysis of these plaques, in addition to their topographic characterization.^21^

Altogether, these data show the importance of a comprehensive assessment by the radiologist of the presence or not (and quantification, whenever possible) of calcification by tomographic study of the chest, since this study demonstrated that the excellent diagnostic capacity of the score, despite the difference between the methods.

### Study limitation

The limitation of the study was the need of administration of a beta-adrenergic blocker, which is not a routine practice in chest CT. We believe that patients with elevated heart rate will present more artifacts in the non-triggered images. Also, it is extremely important that these results could be applicable in other centers, with different scanners, but our results should be tested first for other technologies before being extrapolated.

## Conclusion

The present study showed an excellent correlation between quantification of the calcified plaques by non-ECG-synchronized chest CT as compared with CS in studies using beta-blockers.

Based on our findings, the use of non-ECG-synchronized CT as an alternative method for CS determination should be encouraged, to help in the follow-up and risk stratification of patients who may not have access to cardiac CT.
